# A Retrospective Analysis of the Neurological Evaluation of Cases With Neonatal Hypoglycemia

**DOI:** 10.7759/cureus.31088

**Published:** 2022-11-04

**Authors:** Gurkan Gurbuz, Selen Gur, Sinan Tufekci, Hulya Halis

**Affiliations:** 1 Pediatric Neurology, Namik Kemal University, Tekirdağ, TUR; 2 Pediatrics, Namik Kemal University, Tekirdağ, TUR; 3 Neonatology, Namik Kemal University, Tekirdağ, TUR; 4 Neonatology, Irmet Hospital, Tekirdağ, TUR

**Keywords:** neonatal hypoglycemia, neonatology, outcome, newborn, hypoglycemia, epilepsy

## Abstract

Introduction

Hypoglycemia is a common treatable metabolic disorder in the neonatal period. It can cause several neurological problems if untreated. In this study, the neurological outcomes of cases with hypoglycemia in the neonatal period were examined retrospectively, and the study aimed to determine risk factors and prognostic factors.

Methods

In this retrospective study, cases were followed in the pediatric neurology outpatient clinic between 2019 and 2022, and patients with a history of hypoglycemia in the neonatal period were enrolled and analyzed retrospectively. The laboratory studies and clinical findings of the cases were obtained from follow-up records from the pediatric neurology outpatient clinic retrospectively. Physical examination findings, hypoglycemia levels, and symptoms of hypoglycemia, if any, were obtained retrospectively from the discharge files of the patients.

Results

A total of 70 cases were included in the study. Twenty-eight were girls and 42 were boys. Forty of the cases were preterm. The number of asymptomatic cases was 38, and the number of symptomatic cases was 32. There was no significant difference in regard to the mean lowest serum glucose levels between symptomatic and asymptomatic cases. Thirty-three of the patients had neurological problems such as neuromotor developmental delay, cortical blindness, and epilepsy. Abnormal magnetic resonance imaging (MRI) findings were detected in 30 of the symptomatic cases and eight of the asymptomatic cases. The rate of neurological sequelae in asymptomatic cases was found to be significantly lower than in symptomatic cases.

Conclusions

Patients who have a symptomatic hypoglycemic period, maternal gestational problems, and abnormal MRI carry a high risk for neurological sequelae, and these findings indicate poor prognosis.

## Introduction

Glucose is the most important energy source of cerebral metabolism. In the neonatal period, when transiting from an intrauterine to an extrauterine environment, plasma glucose levels decline deeply when compared to later stages of life. Therefore, hypoglycemia is one of the most important metabolic disorders encountered in the neonatal period. Early recognition and treatment of hypoglycemia are of great importance, as hypoglycemia in the neonatal period may cause irreversible neurological damage such as epilepsy, cortical blindness, and cerebral palsy.

The American Academy of Pediatrics (AAP) guideline recommends that serum glucose levels ​​below 25 mg/dl in the first four hours and below 35 mg/dl in the four to 24 hours should be treated with intravenous glucose, even if the baby is asymptomatic [1]. It is recommended to keep serum glucose levels ​​after 24 hours above the safe level of 50 mg/dl.

Neonates exposed to hypoglycemia may present with many symptoms such as convulsions, irritability, poor sucking, and high-pitched crying. If hypoglycemia is not treated, it causes neurological problems such as cortical blindness, drug-resistant epilepsy, mental retardation, behavioral problems, and neuromotor developmental delay [2]. Neonatal hypoglycemia affects especially the parieto-occipital parts of the brain and neurological problems in neonatal hypoglycemia are due to this central nervous system effect [3].

There is still limited evidence-based consensus about the developmental outcome of patients with neonatal hypoglycemia. This study aimed to find out if these radiological, laboratory, and clinical findings can be indicative of poor development in cases with hypoglycemia and to determine the risk factors and prognostic factors.

## Materials and methods

Patients with a history of neonatal hypoglycemia between 2019 and 2022 were enrolled in the study. The laboratory studies and clinical findings of the cases were obtained from follow-up records of the pediatric neurology outpatient clinic. Those who had a follow-up period of less than three months and did not complete their examinations were excluded from the study.

Definition of hypoglycemia was defined according to the AAP guidelines. Persistent hypoglycemia was accepted as the condition of not regaining the ability to maintain blood glucose levels despite the 48th hour of life and intravenous glucose infusion. Findings such as apnea, convulsion, hypotonia, irritability, and poor sucking were considered symptoms of hypoglycemia. All symptoms and neonatal examinations were performed and evaluated by a neonatologist. Since it was aimed to evaluate the risk factors for neurological sequelae of cases with hypoglycemia, the patients were analyzed by dividing them into two groups as those with and without neurological sequelae.

To determine the etiology of hypoglycemia, urine and serum amino acids, urinary organic acids, serum carnitine profile, blood gas, urine ketone analysis, and serum ammonia level and hormone panels from all patients were retrospectively analyzed from patient files. Brain magnetic resonance imaging (MRI) scans of patients who were thought to have neurological problems were examined by 1.5 Tesla Siemens Magnetom Symphony (Siemens, Erlangen, Germany). MRI results were obtained from patient files and radiology reports.

Genetic analysis was also studied in cases with dysmorphic features and intermittent hypoglycemia. Initial genetic tests were usually karyotype analysis and array comparative genomic hybridization (array-CGH). Whole exome sequencing (WES) was studied from undiagnosed cases.

Statistical analyses were performed using the Statistical Package for the Social Sciences (SPSS) version 20 software package (IBM Corp., Armonk, NY). ANOVA (analysis of variance) and chi-square test were used for the statistical analysis. Local ethics approval was obtained from the Ethics Committee of Namik Kemal University (approval number: 2021.261.11.05).

## Results

The clinical and laboratory characteristics of the patients are summarized in Tables 1, 2. A total of 70 cases were included in the study. Twenty-eight (40%) were girls and 42 (60%) were boys. Forty (57.1%) of the cases were preterm. Of the patients, 65.7% (n = 46) had maternal gestational problems such as preeclampsia, placental insufficiency, and abortus imminens. The most common maternal gestational problem was found to be gestational diabetes mellitus (n = 16). It was thought that the reason for the high rate of cesarean sections in the patients may be due to maternal gestational problems.

**Table 1:** Demographical, clinical, and radiological findings of the patients regarding neurological sequelae.

Parameters	Patients with neurological sequelae (n = 33)	Patients without neurological sequelae (n = 37)	Total	P-value
Mean follow-up duration, month (SD)	10.2 (±2)	6.4 (±1.5)	8.3	0.1
Gestational age				
Preterm, n (%)	23 (32.8)	17 (24.2)	40 (57)	0.08
Term, n (%)	10 (14.2)	20 (28.7)	30 (43)	
Gender				
Male, n (%)	15 (21.4)	27 (38.5)	42 (60)	0.1
Female, n (%)	18 (25.7)	10 (14.2)	28 (40)	
Delivery type				
Vaginal, n (%)	8 (11.4)	12 (17.1)	20 (28.5)	0.04
Cesarean section, n (%)	25 (35.7)	25 (35.7)	50 (72.5)	
Gestational problem				
Present, n (%)	29 (41.4)	17 (24.2)	46 (65.7)	0.03
Absent, n (%)	4 (5.7)	20 (28.5)	24 (34.2)	
Mean lowest plasma glucose level, mg/dl (SD)	20 (±5)	32 (±7)	-	0.07
Persistent hypoglycemia	1 (1.4)	2 (2.8)	3 (4.2)	-
Symptomatic hypoglycemia				
Present, n (%)	25 (35.7)	7 (10)	32 (45.7)	0.01
Absent, n (%)	8 (11.4)	30 (42.8)	38 (54.3)	
Magnetic resonance imaging				
Normal, n (%)	3 (4.2)	19 (27.1)	22 (31.4)	0.01
Abnormal, n (%)	30 (42.8)	8 (11.4)	38 (54.2)	
Non-applied, n (%)	-	10 (14.2)	10 (14.2)	

**Table 2:** Clinical and radiological findings of the patients.

Clinical and radiological features	
Symptomatology in symptomatic hypoglycemic cases (n = 32)	
Convulsion	14
Poor sucking	5
Hypotonia	5
Irritability	3
Tremor	3
Apnea	2
Hypoglycemia etiology (n = 70)	
Gestational diabetes mellitus	16
Asphyxia	12
Feeding problems	10
Infection	10
Metabolic-genetic disorders	4
Unknown	18
Maternal gestational problem (n = 46)	
Gestational diabetes mellitus	16
Preeclampsia	14
Infection	11
Abortus imminens	4
hemolysis, elevated liver enzymes, and low platelet (HELLP) syndrome	1
Abnormal magnetic resonance findings (n = 38)	
Multifocal sequelae of gliotic changes	28
Occipital gliotic changes	7
Total cerebral atrophy	3
Neurological sequelae (n = 38)	
Epilepsy	24
Neurodevelopmental retardation	18
Visual problems	15
Feeding problems	10
Sensorineural hearing loss	2

The number of asymptomatic cases was 38 (54.3%), while the number of symptomatic cases was 32 (45.7%). The most common symptom in the cases was convulsions. There was no significant difference between the mean lowest serum glucose levels of symptomatic and asymptomatic cases. Of the patients, 33 (47.1%) had neurological problems such as neuromotor developmental delay, cortical blindness, and epilepsy. The most common neurological problem was evaluated as epilepsy.

Abnormal MRI findings were detected in 38 (54.2%) cases. The most common MRI pathology was multifocal gliotic areas, which were seen in 28 cases. The rate of neurological sequelae in asymptomatic cases was found to be significantly lower than in symptomatic cases. In some of the patients (n = 20, 28.5%), no pathology that could cause hypoglycemia was found. The most common etiologic cause was gestational diabetes (n = 16, 22.8%). This was followed by asphyxia (n = 12, 17.1%), feeding problems (n = 10, 14.2%), and infections (n = 10, 14.2%), respectively. Persistent hypoglycemia was detected in three patients, and blood sugar regulation was achieved in two of them with glucagon therapy.

Since the main aim of the study was to determine the factors that increase the possibility of hypoglycemia that cause neurological sequelae, patients were analyzed by dividing them into two groups as those with and without neurological sequelae. Neurological sequelae were found to be significantly more common in cases born to mothers with gestational problems and exposed to hypoglycemia (p = 0.003). It was thought that the reason for the high rate of neurological sequelae in babies born by cesarean section was the high need for emergency cesarean section in cases with gestational problems. While the number of groups with and without hypoglycemia symptoms was close to each other, neurological sequelae were found to be significantly higher in symptomatic cases (p = 0.01).

## Discussion

In this study, 70 cases exposed to hypoglycemia in the neonatal period were discussed. These cases were evaluated neurologically and risk factors causing neurological sequelae were focused to be found. As a result of this study, patients with symptomatic hypoglycemia, abnormality in MRI, and with maternal gestational problems were found to be at risk for neurological sequelae.

Many studies on the current subject have obtained different results. In a retrospective study by Çaksen et al., 110 newborns and infants with hypoglycemia were included. It has been reported that pathological MRI was more common in symptomatic infants. Regarding neurological sequelae, “cerebral palsy” and “cerebral palsy with epilepsy” were seen in approximately 45% of patients [2]. In our study, the sequelae rate was found to be 47.1% in parallel with this study. Cortical blindness, neuromotor developmental delay, and epilepsy were frequently seen in hypoglycemia, and these findings have been attributed to the effects of hypoglycemia in the occipital region.

In a study conducted by Yalnizoglu et al., 13 cases with hypoglycemia were examined and lesions were observed in the parieto-occipital white matter in all the patients [3]. This involvement was also defined specifically for hypoglycemia by Barkovich et al. [4]. It has been stated that brain damage due to hypoglycemia takes place specifically in the parietal and occipital lobes due to the increased synaptogenesis and axonal proliferation in this region during the neonatal period [5]. In our study, MRI was required for 60 patients, and 38 of them were found to have a pathology. Seven patients had images specific for hypoglycemia, while the other 23 had multifocal white matter lesions. The reason for the higher incidence of multifocal lesions was thought to be due to the fact that some patients had hypoxic-ischemic encephalopathy and white matter damage due to preterm birth, creating a superposition in the clinical picture.

In our study, there was no statistical difference between the mean lowest hypoglycemia values ​​between symptomatic and asymptomatic cases. The most common symptom was seen as convulsion. The reason for this is that convulsions are too obvious to be overlooked, but hypotonia, agitation, and tremor are more vague findings and may be due to many factors other than hypoglycemia. In our study, hypoglycemia persisted in three patients, and hypoglycemia continued despite maximum intravenous glucose infusion. All three identified patients were babies of gestational diabetic mothers. While one of these patients had neurological sequelae, the other two did not.

A previous study investigated the effect of neonatal hypoglycemia on child development with the CHYLD (Children with Hypoglycemia and their Later Development) study. A total of 528 cases were enrolled and evaluated at the age of two years, and it was determined that there was no developmental difference between the patients who did and did not need hypoglycemia treatment [6]. Qiao et al. detected no abnormality in the examinations performed in the areas of gross motor, fine motor, adaptability, language, and social skills in cases with hypoglycemia, unlike ours [7]. These findings are inconsistent with our study, but in the prospective follow-up of the same patient group, they were re-evaluated at the age of 4.5 years and at school age, and it was found that the academic performance of the cases requiring intervention for hypoglycemia was worse than those who did not [8,9]. This information emphasizes the importance of long-term follow-up in cases with neonatal hypoglycemia who may experience learning problems at school age even if there is no motor deficit.

In our study, genetic and hereditary metabolic diseases were found in four patients. Adrenal insufficiency due to proopiomelanocortin (POMC) gene mutation was found in one infant, glycogen storage disease type 1 in one patient, glycogen storage disease type 3 in one patient, and Beckwith-Wiedemann syndrome in one patient. These diseases are all associated with hypoglycemia in the literature [10-13]. Although the hypoglycemic periods of two patients diagnosed with glycogen storage disease were not resistant, albeit it was observed that it appeared intermittently after the neonatal period. The patient with POMC mutation also showed typical features of the disease with dysmorphic features, adrenal insufficiency, and red hair. Patients diagnosed with Beckwith-Wiedemann syndrome showed classical findings of the syndrome like macrosomia, omphalocele, macroglossia, and hypotonia. These four cases are 4.7% of patients. Although the detection rate of genetic and hereditary metabolic diseases is low, metabolic screening is recommended for all hypoglycemic patients to detect treatable causes.

A prospective study by Wickstrom et al. reported that neuropsychiatric diseases such as autism and attention deficit and hyperactivity disorder are more common in later ages in cases with hypoglycemia in the neonatal period than in cases without hypoglycemia [14]. In our patient group, no patient with signs of autism was found. We have collected early outcomes of the patients who had neonatal hypoglycemia but long-term follow-up is recommended for the neurocognitive outcomes. Neuropsychiatric follow-up of the patients is still ongoing.

This study has two main limitations. The first limitation is the small number of participants. We think that there will be more patients diagnosed with genetic and metabolic disorders in multicenter studies with the large patient group. The second limitation is the short duration of follow-up periods of the patients. As we mentioned before, we have only reported the early outcomes of the cases. But long-term outcomes and cognitive investigations are very important in this group of patients.

## Conclusions

It is important to follow up on the cases with neonatal hypoglycemia in terms of neurological sequelae. In symptomatic cases, those with maternal gestational problems and those with MRI lesions are at risk for the development of neurological sequelae. Considering that cases with symptomatic hypoglycemia have a poor prognosis, it is of great importance to perform a complete neurological examination of these patients at the time of diagnosis in terms of neurodevelopmental follow-up. In addition, it should always be kept in mind that the evaluation of neonatal hypoglycemia cases in terms of metabolic and genetic diseases is important for the early diagnosis and treatment of patients.
